# Community Gardening: Stress, Well-Being, and Resilience Potentials

**DOI:** 10.3390/ijerph17186740

**Published:** 2020-09-16

**Authors:** Way Inn Koay, Denise Dillon

**Affiliations:** 1 Department of Psychology, James Cook University Singapore, Singapore 387380, Singapore; koay.way.inn@sgh.com.sg; 2 Department of Psychology, Singapore General Hospital, Singapore 169608, Singapore

**Keywords:** community gardening, contact with nature, connection to nature, mental health promotion, well-being, stress, resilience, self-esteem, optimism, openness

## Abstract

The financial and health burdens of stress associated with increased urbanization have led to a demand for mental health enhancement strategies. While some extant literature details mental health benefits of community gardening, a coherent narrative on the construct of resilience and its relationship with the mental health benefits of community gardening is lacking. The present study examined the relationship between community gardening and a number of mental health benefits, in the forms of subjective well-being, stress, resilience potentials, and resilience factors (self-esteem, optimism, and openness). A total of 111 residents in Singapore completed a survey. Results from Multivariate Analysis of Covariance (MANCOVA) and Pearson’s correlation analyses show that, after controlling for age and levels of connection to nature, community gardeners reported significantly higher levels of subjective well-being than individual/home gardeners and non-gardeners, indicating that engagement in community gardening may be superior to individual/home gardening or non-gardening outdoor activities. Community gardeners reported higher levels of resilience and optimism than the non-gardening control group. These novel results indicate some potential for mental health benefits in urban environments, specifically in terms of subjective well-being and resilience. These findings have implications for future research in clinical psychology, mental health promotion, and policy.

## 1. Introduction

It is commonly accepted that stress varies on a continuum from maladaptive responses to acute or enduring anxiety to more severe stress reactions that negatively impact general well-being and daily functioning, depending on individual vulnerability and resilience potentials. Abundant research findings have evidenced that stress is associated with poor mental and physical health (e.g., depression, cardiovascular disease) [1,2,3]. The cumulative effects of daily stressors are also found to be an important predictor for the emergence of depression, which is found to be the most common mental disorder in Singapore with a lifetime prevalence of 5.8% [4]. Stress has also been found to be associated with overall lower well-being [5] and incurs costs on society [6,7]. Hence, there is an ongoing need for low-cost and effective strategies to enhance mental health by lessening the impact of stress or promoting well-being, particularly in Singapore.

Recent shifts in societal views and research findings have shown that community gardens have ample potential to provide spaces for individual and communal good, and to contribute to community bonding, health and urban-environmental equilibrium that many in Singapore and the world are yearning for. Consequently, there is a rise in the emergence of community gardens across cities, including Singapore. Congruent with the evolution of the National Parks of Singapore’s (NParks) vision of Singapore from “Garden City” to “City in a Garden” [8], the Singapore government has allocated substantial funding for a range of initiatives. These include establishing world-class gardens, optimizing urban spaces for greenery, enhancing community gardening movements, and incorporating community gardens as vital aspects of urban design and city planning [9,10]. The Community in Bloom (CIB) initiatives, launched in 2005 by NParks [11], have fostered nearly 1000 community gardens nationwide and inspired a community spirit amongst residents to co-achieve Singapore’s “City in a Garden” vision. The funding is distributed for the maintenance of the community gardens and for horticultural assistance. Given the substantial funding invested, it is important to determine if the current Singapore community garden movements have the potential for sustainability and to identify their effects on the mental health of residents in Singapore. Hence, the existing literature on the benefits of connection to nature and gardening activities, in combination with current data on community gardening activities, needs to be examined to demonstrate the patterns of contribution (e.g., in terms of nature or social elements) of community gardening in Singapore in a clearer way. While literature on the benefits of community gardening in Western countries (e.g., U.S.A. and U.K.) is available, there is a scarcity in the literature focusing on benefits stemming from community gardening activities in Eastern-oriented countries, such as Singapore.

In view of the significant health and financial burdens of stress and depression on the individual and society, the substantial funding invested in community garden initiatives, the potential benefits of community gardening to mental health, as well as the scarcity in the research literature specific to Singapore, the current study is both relevant and timely.

As a beginning, evidence of the effects of natural environments (or green space) and gardening activities on mental health is first reviewed. Subsequently, the focus is shifted to community gardening alone, followed by a review of the restorative properties of natural environment and a discussion of existing evidence of the effects of community gardening on mental health (e.g., well-being and stress). The final section evaluates the future prospects and other potential benefits of community gardening by analyzing its relationships with resilience and resilience factors respectively. The literature review helps shape the hypotheses critical to answering specific research questions.

### 1.1. Restorative Properties of Natural Environments

Two theories have been expansively cited in the literature to underpin the associations between the restorative properties of natural environments and recovery from stress and mental fatigue, suggesting mental health benefits—the psycho-physiological stress reduction framework [12] and the Attention Restoration Theory (ART) [13,14].

According to the psycho-physiological stress reduction framework [12], humans are biologically attuned through evolution for immediate positive responses to safe, natural environments associated with survival, such as trees, vegetation, and water. Thus, exposure to natural stimuli can support restoration from stress, which includes recovering from markedly high or low physiological and psychological conditions and recharging of the energy consumed in response to stress [15]. Exposure to natural environments can also mediate the destructive effect of stress by reducing negative mood while enhancing positive affects [15,16]. This restoration-from-stress effect can be gained through the activity of gardening, and this is empirically supported by the results of a field experiment in which mood was found to be restored, and stress levels, as assessed by cortisol levels, were found to reduce with the strongest extent following gardening, as compared to a control group [17]. A longitudinal survey with empirical data also indicated that adults who moved to greener residential areas benefitted from sustained improvements in their mental health, supporting the restorative effects of natural environments [18].

Meanwhile, according to the ART [14], humans have a predisposition to respond positively to the contents and features of natural environments (e.g., green landscapes) and have a preference for scenes depicting natural rather than urban environments because of the cognitive restorative effect. Berman, Jonides, and Kaplan [19] employed an experimental design to review the cognitive benefits of interactions with natural environments compared to urban environments. The improved directed-attention abilities after interacting with nature validated the ART. The cognitive restorative effect, as evidenced by improved performance on tasks involving attention and cognitive processing following visual exposure to natural environments [20,21], helps to restore an individual’s voluntary or directed attention, which in turn reduces mental fatigue. By reducing mental fatigue that manifests itself in negative emotions, the probability that individuals experience a stress response due to cognitive overload is reduced simultaneously.

Both theories highlight that exposures to natural environments are more restorative (e.g., provide physiological, emotional and attention restoration) than urban environments [22]. Inability to restore attentional capacity aggravates the mental fatigue state and can also threaten well-being and mood, as well as affecting work performance and interpersonal relationships. Thus, individuals who are deprived of exposure to natural environmental features will exhibit behaviors caused by mental fatigue.

### 1.2. Effects of Natural Environments and Gardening Activities on Mental Health

Humans depend both physically and emotionally on nature [23], and both spending time in nature and connection to nature were observed to offer a number of cognitive, effective, and physiological benefits [24]. Measures such as the Inclusion of Nature in Self (INS) scale [25,26], the Connectedness to Nature scale [27], and the Nature Relatedness scale [28] all capture expressions of the same construct: a subjective connection to nature [29,30]. Exposure and connection to natural environments not only has direct benefits through stress recovery and mental fatigue restoration, it also has implicit benefits by serving as a buffer against stressful life events [17]. Regular contact with nature is also found to lead to long-lasting, positive impacts on mental health, including a reduction in depressive and anxiety symptoms [31]. Landscapes consisting of natural elements (e.g., groves of pine) have been found to evoke feelings of pleasure and calmness, and are conducive to restoration from stress [32]. Green spaces in urban environments are associated with stress relief [33] and longevity for senior citizens [34]. On the other hand, stress has been found to be a mediator of the relationship between neighborhood greenery and mental health [35]. In addition, loneliness, perceived decreased social support, and stronger social cohesion have been shown to partially mediate the relationship between greenery and mental health [35,36].

Established evidence has supported the benefits of gardening activities, one form of exposure to natural environments. Kaplan [37] initiated groundbreaking research by employing interview and questionnaire approaches to investigate the psychological benefits of home/individual gardening and community/plot gardening. The findings show that gardeners rated gardening as a valuable means by which to spend time, relax, and gain a sense of accomplishment. Subsequently, gardening activities have been widely studied as potential methods of restoration from stress and negative mood, or as therapeutic interventions (e.g., horticultural therapy) for people suffering mental health-related difficulties (e.g., [38,39,40]). Significant reductions in stress, fatigue and depressive symptoms in the context of gardening activities have also been reported [41,42]. Recent meta-analytic research documents considerable evidence for the positive effects of gardening on both physical and mental health, after adjusting for publication bias [43]. Increasing research reflects a broader interest in the role of different forms of gardening activities (e.g., home gardening, community gardening) in enhancing mental health respectively.

### 1.3. Community Gardening

Community gardening generally refers to activities ranging from multiple individual plots to collective cultivation at a common space—a school or a hospital, in a public space or a neighborhood. The origin of community gardening in the United States can be traced to the late 1800s when abandoned gardens were opened to the urban unemployed to grow food [44]. The allotment garden is a sub-type of the more general category of community garden and its origins can be traced to Europe in the 18th century when parcels of land were open to deprived plot holders and their relatives to cultivate food [45]. In Singapore, community gardens are described by the National Parks Board [46] as “common spaces where people of different demographics come together to create, develop and sustain a gardening space in their locality”.

Community gardening, a form of exposure and connection to natural environments, emerges as a complex multi-factorial activity that involves collective effort by the community to cultivate plants. Community gardening is recently getting much more research attention because of its direct and indirect impacts on individual mental health and its potential contribution to community bonding and building a sense of community [47]. It benefits the gardeners themselves and the broader community by supplying food, high-quality nutrition, recreation opportunities, and physical activities within neighborhoods, offering a way to restore connection with nature, and contributing to restoration of ecological connection [44,48,49,50]. Community gardening has been reported to have positive effects on mental health due to the direct contact with natural environments it affords to community gardeners and its tranquil, restorative, and social nature [47,51,52]. Gardeners described involvement in a community garden at Alabama as stress-relieving and relaxing, suggesting mental health benefits [53]. Community gardening has also been discussed as a further mechanism to explain beneficial effects from nature for coping with daily stress [54]. Community gardeners reported that they valued the garden as a personal achievement, suggesting community gardening is associated with pride and well-being [53]. Lovell, Husk, Bethel, and Garside [55] recently conducted a mixed method systematic review and this study is the first systematic review, which has highlighted the health and well-being benefits of community gardening for children and adults. They suggested the mechanisms and different levels of operation through which community gardening may impact health and well-being. Overall, the benefits of community gardening on individual mental health and community cohesion are evident.

Empirical research studies with quantitative data on the mental health benefits of community gardening are relatively scant. Two empirical studies show that participants reportedly gained psychological benefits relating to quality of life including reduced stress levels, improved self-esteem, and increased social interaction, after taking part in community gardening [51,56]. In a study conducted by Wakefield, Yeudell, Taron, Reynolds, and Skinner [57], which employed participant observation and in-depth interviewing approaches to collect data, the findings showed that community gardeners perceived the community garden as a place offering positive social interaction, improved mental health and increased physical activity, suggesting associations between community gardening and community cohesion. The findings of a recent study in China indicated that participation in a community gardening benefits people’s mental health experience [58], which likely contributed by the improved social exchange among community residents and proximity to nature. A limitation of the study is that mental health was measured with only two items and mental health benefits were not explored in specific forms.

### 1.4. The Relationship Between Community Gardening and Individual Constructs

Under the perspective of policy makers, researchers, or community gardeners, the benefits of community gardening are commonly categorized into a more general and unified category—the mental health benefit. Here we review and distinguish the individual, specific features within the mental health benefit.

#### 1.4.1. Well-Being

The construct of well-being and the proposed pathways to its attainment have generally been conceptualized according to two philosophical perspectives: hedonia and eudaimonia [59,60]. Past dispute pertaining to the pursuit of well-being culminated in an agreement that both hedonic and eudaimonic perspectives are divergent and contribute to well-being in distinctive manners [61]. Based on the hedonic perspective, well-being is composed of one’s perceptions of pleasantness and is attained though the maximization of pleasurable moments and the satisfaction of one’s desires [62]. The hedonic perspective considers well-being as subjective evaluations about the quality of one’s life, which, in turn, are translated and operationalized into the construct of subjective well-being [63]. Subjective well-being consists of an affective component with higher prevalence of positive emotional experiences over negative emotional experiences, and a cognitive component focusing on a personal judgment on the satisfaction level with life and with specific life domains respectively [63,64]. Meanwhile, based on the eudaimonic perspective, well-being is about pursuing individual values or meanings in life and is attained through fulfilling an individual’s inherent potential for personal growth [65].

The literature on well-being is broad, and the construct of subjective well-being has been extensively studied. Of interest to the current study is the recent resurgence of interest in contact with natural environments and its ties with well-being [66,67]. Studies have recorded that contact with natural environments and connection to nature are associated with affective well-being, such as enhancing happiness [68,69,70,71,72]. A meta-analysis even proposed quantitative evidence on the positive links between nature connectedness and happiness as an important affective component of subjective well-being [29]. Since gardening is one of the conventional approaches of interacting or connecting with nature, there is an increasing slant towards research that examines the links between gardening activities and happiness, or subjective well-being. Horticultural and physical activities, such as gardening have been investigated and found to result in positive effects in terms of well-being and self-esteem [73]. A pilot pre- and post-test study conducted by Heliker, Chadwick, and O’Connell [74] documented an improvement in subjective well-being, feelings of satisfaction and spiritual well-being of elderly participants (age range 63–90) as a consequence of involvement in a gardening project comprising twelve classes covering educative topics like propagation techniques, terrariums, and planting herbs. However, the limitation of this study is the lack of a no-treatment control group for the comparison of the effects of gardening. In a survey study, home gardening was found to be positively correlated with improved mental health and well-being [75]. Adolescents who did home gardening reported fewer depressive symptoms and improved emotional well-being than those who did not do gardening.

Both home gardening and community gardening include the elements of connection to nature, and gardening experience. Community gardening is different from home gardening because of its additional social element as well as its gardening locations [76], and gardening with others often leads to increased socialization and responsibility to the group [77]. Thus, there is a shift towards investigating the importance of community gardening in maintaining or enhancing subjective well-being that involves the preponderance of pleasant affect over unpleasant affect [63]. Given that social relatedness has been found to predict happiness or subjective well-being [78], it is reasonable to expect that community gardening should have at least comparable effects to home gardening on subjective well-being.

Milligan, Gatrell, and Bingley [79] conducted a study employing primarily ethnographic methods and found that communal gardening activities enabled older community gardeners (aged over 60 years) to acquire subjective well-being in the forms of a sense of achievement, pleasure, satisfaction and enhanced quality of life. Most of the older adults opted to garden communally with others on the allotment sites, in turn reducing social isolation that can serve as a buffer to stressors. The study results have supported the potential, specific benefits of communal gardening over and above the contribution of the traditional ‘isolated’ gardening to health and well-being of older adults. Austin, Johnston, and Morgan [80] conducted a pilot study employing a one-group (age range 57–78 years), pre-test/post-test design to examine the effects of participation in a community gardening program on the participants’ functional health, depression, and physical fitness. Their results indicated a decline in negative emotions and distress following gardening activities.

Kingsley, Townsend, and Henderson-Wilson [81] employed a qualitative semi-structured interviewing approach to elicit responses from community gardeners (age range 20–69 years) in order to have greater insight into their understanding and perceptions of health and well-being benefits following their engagement on the ‘Dig In’ community garden in Melbourne. The community gardeners described that they experienced enhanced well-being because the community garden offers a sense of achievement, enables them to feel closer to nature and offers a getaway from daily stresses. Van den Berg et al. [17] conducted a survey among gardeners with a mean age of 59.6 years from 12 allotment sites in the Netherlands and their neighbors as the control group, and the survey questionnaires included questions about their health, well-being with four components (i.e., amount of stress, life satisfaction, loneliness, and social well-being) and physical activity. The results further support the contribution of community gardening activities on well-being, as evidenced by the higher scores of the older gardeners on health and subjective well-being measures compared with those of the control group. A case-control study showed that 136 gardeners with a mean age of 55.8 years who engaged in allotment or community gardening reported higher levels of mood and self-esteem, and a reduced level of psychological distress as compared with 133 non-gardeners [82].

In summary, these research findings imply that community gardening has positive benefits on gardeners’ mental health through a contribution to the different aspects of well-being, such as increased levels of positive affect and improved life satisfaction.

#### 1.4.2. Stress

Accumulating research studies supply evidence for the benefits of community gardening on stress rejuvenation and well-being. The construct of stress can be defined as difficult life events or chronic hassles and stressors that are non-pleasant to the extent of threatening the well-being and existence of the respective individual [83]. Higher relaxation is also deemed a vital component of well-being [84]. Perceived stress can also refer to a composite measure of the extent and amount to which one’s life situations are appraised as stressful [85].

Similar to the construct of subjective well-being, the construct of stress has been extensively studied. Of interest to this study is the established finding that stress and adverse life events negatively impact mental health, and many studies document that stress precipitates negative psychological symptoms [86,87]. In view of the negative effects of stress, much research has been dedicated to discover adaptive and effective coping strategies for stress. The beneficial effects of nature on stress have been examined thoroughly, and visiting gardens, which provide exposure to nature, has been advocated as a perceived stress reduction strategy [83]. Research findings indicate that access to green spaces has been associated with restoration from stress, greater social capital, more positive emotions, and an increased sense of well-being [88].

The impacts of community gardening on mood and stress are highlighted by a study evaluating the community gardening programs at some of California’s domestic violence shelters [89]. Stuart employed survey and structured interviewing approaches with 81 program participants. Her findings show that gardening eased adjustment to the domestic violence shelter and relieved stress upon seeing new growth of plants. In the study by Wakefield et al. [57], the community gardeners indicated merely being present and belonging to a community garden helped relieve stress, as evidenced by quotes such as: “… sometimes when you are stressed out… when you go to the garden, you feel different.” (p. 97). This point was echoed in a study conducted by [81] in which a community gardener indicated that the “garden has a lot of things going for people who are in stressful environments of today and who want to get away from these pressures.” (p. 211). A study in Australia applying semi-structured interviews also showed that stress relief was one of the motivations for community gardening participation [90], but such qualitative insights need to be supported by quantitative data.

Hawkins, Thirlaway, Backx, and Clayton [91] conducted a cross-sectional study to investigate the contribution of communal gardening activity at an allotment site on the perceived stress amongst participants (age range 50–88 years). The participants were categorized into four groups with different levels of physical activity and contact with nature: allotment gardeners, home gardeners, participants who performed outdoor physical activity via health walks and participants who performed indoor physical activity. The results showed significantly lower levels of perceived stress for the allotment gardeners than participants who performed indoor physical activity. However, there was no significant difference in the perceived stress levels between the allotment gardeners and home gardeners, or those who performed outdoor physical activity.

#### 1.4.3. Resilience

Due to the shift from the “problem-oriented” paradigms of psychopathology toward “strength-based” paradigms where the focus is on adaptive coping despite stress and adversities, researchers subsequently bring the construct of resilience into the spotlight by investigating the resilience-stress relationships and well-being. Many studies propose that, when individuals experience stressful life events, their positive assets such as trait resilience and self-efficacy can be activated to support them for successful adaptations and active coping [92,93,94]. Perceived stress has also been illustrated to have a negative correlation with resilience [95]. As adaptive coping is associated with mental health [96], it is relevant to review the construct of resilience and its relationship with community gardening in order to get a clearer picture of the mental health benefits of community gardening.

Literature has demonstrated that resilience is broadly acknowledged as a multidimensional construct with a few common fundamental components amidst the diversity [97,98,99,100,101]. The construct of resilience can be employed to illustrate the ability to bounce back from stress to optimal levels of well-being [102,103]. Alternatively, resilience refers to the ability to enable individuals to adapt to hardships or the ability to enable individuals to adapt well to stressful situations [104,105] and the ability to deal with shocks and unexpected changes [106].

Windle [107] employed the integrated methods of systematic review, concept analysis, and face-to-face consultation to offer a reality-driven perspective of attributes and consequences of resilience. According to Windle, resilience is defined as follows:

A process of negotiating, managing, and adapting to significant sources of stress or trauma. Assets and resources within the individual, their life, and environment facilitate this capacity for adaptation and ‘bouncing back’ in the face of adversity. Across the life course, the experience of resilience will vary (p. 163).

Results from past and recent advances of research indicate that individual resilience is connected with multiple mental health-related outcomes such as self-reported levels of stress, burnout, and general indicators of well-being [108]. The moderating effect of resilience in the links between stressful life events and late-life depression appears to be such that resilience could perform as a buffer against the detrimental mental health impacts of stressful life events [109]. Given the resilience-stress relationships, and that natural elements are associated with the reduction of mental fatigue and psychological stress, engagement in community gardening should also be helpful in increasing resilience from stress and leading to significant improvements in well-being. However, published research investigating the relationship of community gardening with the construct of resilience is very scarce. A few recent studies suggest it may have potential but most of the relevant literature has been covered under the umbrella of natural environments and green spaces.

Community gardening has been proposed as a means to foster good health and well-being by furthering resilience on three levels (individual, social, and natural environment), strengthening social resilience, and motivating the execution of other neighbourhood improvements, particularly in deprived areas [110,111,112]. Okvat and Zautra [112] reviewed and summarized the relevant evidence for the cognitive, emotional, social network, economic, climate change mitigation and environmental benefits of community gardening to clarify the role of community gardens in promoting resilience in social ecological systems or the Earth community.

The findings of the study conducted by Chawla, Keena, Pevec, and Stanley [113] extend understanding about the value of community gardening and resilience. They uniquely employed ethnographic observations and interviewing approaches and recruited participants across six study sites covering primary schools and secondary schools. In particular, they conducted interviews with the teen gardeners at two of the six study sites about their gardening experiences in their schoolyards or in an after-school gardening program in which they grew vegetables and herbs collectively, consistent with community gardening activities. The teen gardeners reported feeling relaxed, calm, and peaceful in the midst of and after gardening and provided reasons. Consistent with the past research findings, the results also document that access to nature reduces depression rates and facilitates coping with stress as these participants had numerous chances to expose themselves to positive sensory experiences, and immerse themselves in productive, creative, and cooperative activities. They also find that the natural areas motivated sustained attention and facilitated the development of supportive social relationships, which are an important protective factor for resilience. Thus, it appears that contact with nature created circumstances for enhancing resilience through strategies advocated by Masten and Reed [111]: building assets (e.g., increasing sense of competency and concentration that contribute to self-esteem), reducing risks (e.g., inattention), and mobilizing adaptational systems (e.g., cooperative friendships). The overall study findings reported by Chawla et al. [113] connect stress, resilience, and contact with nature as evidenced by the potentials of green schoolyards in reducing stress and enhancing protective factors for resilience in children and adolescents. However, as two of the study sites involved more active community gardening activities compared to the other sites merely with exposure to nature and green schoolyards, research work in further teasing apart the contributions of connection to nature, gardening experiences, and physical or social elements that community gardens possess is required. A literature gap is identified and further work may be required to focus on the different elements of community gardening and their impacts on resilience respectively.

### 1.5. The Relationship Between Community Gardening and Resilience Factors

Resilience is also found to be fostered with protective factors: individual attributes and qualities and their context, and the relationships with the situations or experiences that predict good adaptation following stress and adversity [114,115]. As a result, resilience can be enhanced through strategies that serve to strengthen individual resilience factors and build assets (e.g., increasing individuals’ concentration that contribute to their self-esteem), reduce risk factors, and mobilize adaptational systems (e.g., friendships and emotional support) [111]. Windle, Bennett, and Noyes [107] summarized that resilience is predominantly measured from a multi-level perspective constituted by several key dispositions and factors that influence individuals’ vulnerabilities to risk, stress, and adversities. Some of the common resilience factors and key dispositions are acceptance of change, optimism, self-esteem, personal competence, and social competence. Previous studies also recorded the associations between factors like optimism, self-esteem, self-efficacy, and extroversion, and fewer negative consequences after stress and traumatic events [116]. Moreover, optimism, the ability to appraise stressful events in less intimidating ways and the capacity to reframe adverse experiences in a positive way have each been found to link with resilience [117,118]. Likewise, Storm and Rothmann [119] found that openness is correlated with positive re-appraisal of stressful situations and acceptance of stressors. Openness to experience is also found to be associated with problem-solving capacity and creativity, which are deemed integral to resilience [120]. The resilience factors investigated in the present study are self-esteem, optimism, and openness.

#### 1.5.1. Self-Esteem

Self-esteem is a psychological construct that reflects one’s overall appraisal of one’s value or self-worth [121]. There is plenty of literature investigating the relationships between self-esteem and subjective well-being and depression [122]. Contact with nature and gardening have been found to have a positive influence on self-esteem, one of the resilience factors [123,124,125,126,127]. However, Freeman et al. [123] did not distinguish between home and community gardening as separate aspects, and how they individually influence self-esteem. The research findings of Hoffman et al. [125] indicate that students who completed a sixteen-week gardening program reported elevated levels of self-esteem and self-efficacy. However, their study was limited by the possibility of biased results originating from a lack of pre-test or baseline condition. Nonetheless, these findings lay the groundwork, which sets the discussion about the association of community gardening with self-esteem. Scott, Masser, and Pachana [128] employed a survey approach to gather qualitative and quantitative data of older adult gardeners (age range 60–90) from various seniors’ groups and a community gardening group. The qualitative data were assessed by deriving themes using Leximancer text analysis software and the results show the psychological and physiological benefits of regular connection with nature through gardening activities, in their own homes or in community gardening groups. The gardening activities were found to provide the gardeners with the possibility of meaningful activity, which in turn is associated with improved self-esteem and sense of achievement. Again, the participants are clustered collectively under the bigger and more general umbrella of a gardening group, and the mechanisms by which home gardening and community gardening individually influence self-esteem remain unknown.

Only one study appears to have investigated how community gardening individually influences self-esteem. A case-control study investigated the well-being and health benefits of 269 participants, half of whom were community gardeners from 10 allotment sites in England [82]. The participants were requested to complete a questionnaire about their self-esteem, mood, and general health. Strength of this study was the inclusion of non-gardeners as a comparison control group. The findings indicated that one session of allotment gardening, which is a subtype of community gardening, can give rise to higher levels of self-esteem and mood, regardless of the duration participants spent on gardening. Compared to the non-gardener control group, the allotment gardening group had higher levels of self-esteem and mood, and a reduced level of psychological distress. Nonetheless, further in-depth work may need to be done to investigate how community gardening solely links with self-esteem.

#### 1.5.2. Optimism

Optimism is a construct defining as a generalized inclination to expect and anticipate positive outcomes in one’s life or future events, with pessimism at the opposite end of the spectrum [129]. People who are optimists tend to report higher subjective well-being because they deal with critical life situations more adaptively than pessimists do [130]. Optimism may also confer resilience to stressful life events, which are related with a risk of mental disorders [131]. Positivity about the future outlook relating to optimism seems to afford individuals an element of resilience when faced with adversity [132].

As engagement in community gardening has been found to influence self-esteem, which is one of the resilience factors, community gardening may have an association with other resilience factors closely linked with self-esteem, such as optimism. To date, only two studies have investigated the link between gardening and optimism. Waliczek, Zajicek, and Lineberger [133] documented that gardeners responded more positively on statements pertaining to optimism and physical self-concept when compared to responses of non-gardeners. Sommerfeld, Waliczek, and Zajicek [134] employed the Life Satisfaction Inventory A to investigate participants’ perceptions of life satisfaction and physical activity level. Their results also showed that gardeners gave significantly more positive answers on the quality-of-life statements (e.g., optimism) when compared with non-gardeners. However, as described above, the literature did not distinguish between home (individual) and community (social) gardening as separate aspects, and how they separately influence optimism. Hence, further work may need to be done to investigate how community gardening individually associates with optimism.

#### 1.5.3. Openness

Openness is considered as the broadest and most contentious personality trait of humans, under the Big Five personality dimensions [135]. Openness is sometimes referred to as change acceptance or positive view of changes [136], and openness to change values were found to be positively associated with the openness to experience trait [137]. Over the years, numerous labels for “openness” have been posited. The openness to experience label originated from the work of Costa and McCrae [138], who found that measures of imagination, intelligence and openness to change had tendencies to co-change. Openness to experience has since been one of the more popular labels capturing “openness” [135,139]. Research findings have documented positive associations between openness to experience and resilience across populations. In addition, a meta-analysis [140] documented a close relationship between openness to experience and the subjective well-being facets of happiness, positive emotions, and quality of life.

Openness to experience has also been found to be associated with connection to nature, which is one of the elements of community gardening [30,141]. This is not an unexpected finding because change is a constant force in natural environments. As gardening involves change and growth in the cultivation of plants, people who enjoy community gardening should likely have more opportunities to be familiar with experiences of change as either growth or decline. Given that higher levels of acceptance to change are found to have associations with high levels of self-esteem and optimism that are resilience factors [142], people who enjoy community gardening may have more acceptance and more openness to life change, which in turn could enhance their resilience potentials.

Further work on the associations among openness, resilience, and community gardening will be a valuable contribution to the existing literature, in view of the fact that these associations specific to community gardening have yet to be explored.

### 1.6. Rationale and Significance of the Present Study

While there has been an increase in popularity in Singapore of community gardening, there is also an increase in published research related to the mental health benefits associated with this activity. However, much of the available research is qualitative and descriptive [143], which left a gap to be filled through quantitative studies. The preceding sections have highlighted several factors and mechanisms of well-being, stress, and resilience associated, respectively, with contact or connection to nature, and different forms of gardening activities, such as home gardening or community gardening activities. While research literature on the adaptive value of community gardening on well-being and stress is available, there is a scarcity in the research literature highlighting the implications of community gardening on resilience. Besides that, the present understanding of the difference between community gardening and individual or home gardening, as well as the contributions of the contact or connection to nature and social elements of the community gardening, is still poor despite the marked consequences of these two elements on well-being and stress respectively. Given the pivotal role of community gardening in the potential for restoration from stress and other mental health benefits, there is a need to understand these questions with greater depth. Thus, the current study was designed to examine the effects of community gardening through the resilience perspective.

Moreover, as engagement in community gardening has been found to be associated with self-esteem, which is one of the resilience factors, community gardening may have an association with psychological phenomena that are closely linked with self-esteem and resilience, such as optimism. Further work may need to be done to investigate how community gardening individually associates with optimism, in order to further supplement the existing literature. With the significant positive associations between openness to experience and psychological resilience, further work on the associations between openness and community gardening will be a valuable contribution to the existing literature. The present study aims to gain insight into resilience in the context of community gardening, by also examining resilience factors such as self-esteem, optimism, and openness.

Furthermore, there is a deficiency in knowledge about the benefits of the respective elements of community gardening, with much of the existing evidence depending on qualitative data. To date, a query that is still under debate is whether the reported well-being and stress reduction benefits are attributed to the exclusivity of respective individual elements of community gardening such as the provision of the physical activity, connection to nature, gardening experience or social elements, or the combination of all these elements. Several systematic reviews have documented the importance of physical activity or exercise for mental health [144,145,146]. However, it is unknown if the presence of physical activity and social elements in community gardening is the core factor that enables community gardening activities to enhance mental health, or if interaction with natural components of the environment is also implicated in positive effects. Thus, research focused on further teasing apart the contributions of these elements of community gardening on the mental health benefits is worth exploring.

Thus, we conducted a cross-sectional type of survey to extend previous studies to examine the implications of community gardening on subjective well-being and stress, as well as to fill in the literature gaps to provide finer clinical understanding and application of community gardening on resilience potentials, along with consideration of the potential contribution of different elements of community gardening.

We will now discuss the aims and hypotheses of the present study.

### 1.7. Aim

The aim of the study is to examine the relationship between community gardening and a number of mental health benefits, in the forms of subjective well-being, stress and resilience as well as the respective resilience factors, along with consideration of the potential contribution of physical activity, connection to nature, gardening experience, or social elements, or the combination of all.

#### Hypotheses

Gardening activities typically involve either a social aspect (community gardening) or individual aspect (individual/home gardening), and studies examining differences between groups usually benefit from the inclusion of a control condition. From existing literature, it was predicted that community gardeners, individual/home gardeners and a non-gardening (outdoor activity) control group would differ on the measures of subjective well-being and perceived stress depending on their variations in the gardening experience or social elements. In particular, it was predicted that community gardeners, as compared to individual/home gardeners and non-gardeners (control), would report higher levels of subjective well-being and lower levels of perceived stress, as a result of the combined effects of the elements of physical activity, gardening experiences, contact or connection to nature as well as social interaction. However, there has yet to be consistent and conclusive evidence on the difference in the levels of resilience and resilience factors across community gardening, individual/home gardening, and non-gardening control groups. Therefore, non-directional hypotheses were then adopted. This current knowledge together with indications of existing gaps in understanding led to the formulation of a set of hypotheses as follows.

**Hypothesis 1** **(H1).** *Community gardeners would report significantly higher levels of subjective well-being than the individual/home gardeners and the non-gardening control group.*

**Hypothesis 2** **(H2).** *Community gardeners would report significantly lower levels of perceived stress than the individual/home gardeners and the non-gardening control group.*

**Hypothesis 3** **(H3).** *Resilience levels would be significantly different across the community gardeners, the individual/home gardeners, and the non-gardening control group.*

**Hypothesis 4** **(H4).** *The levels of the respective resilience factors (i.e., self-esteem, optimism, and openness) would be significantly different across the community gardeners, the individual/home gardeners, and the non-gardening control group.*

**Hypothesis 4a** **(H4a).** *The levels of self-esteem would be significantly different across the community gardeners, the individual/home gardeners, and the non-gardening control group.*

**Hypothesis 4b** **(H4b).** *The levels of optimism would be significantly different across the community gardeners, the individual/home gardeners, and the non-gardening control group.*

**Hypothesis 4c** **(H4c).** *The levels of openness would be significantly different across the community gardeners, the individual/home gardeners, and the non-gardening control group.*

**Hypothesis 5** **(H5).** *Resilience would be positively correlated with subjective well-being.*

**Hypothesis 6** **(H6).** *Resilience would be negatively correlated with perceived stress.*

**Hypothesis 7** **(H7).** *Resilience would be positively correlated with each of the individual resilience factors (i.e., self-esteem, optimism, and openness).*

**Hypothesis 8** **(H8).** *Community gardeners would report significantly higher levels of connection to nature (i.e., the INS score) than the individual/home gardeners and the non-gardening control group.*

**Hypothesis 9** **(H9).** *Connection to nature (i.e., the INS score) would be positively correlated with resilience.*

**Hypothesis 10** **(H10).** *Connection to nature (i.e., the INS score) would be positively correlated with each of the individual resilience factors (i.e., self-esteem, optimism, and openness).*

## 2. Materials and Methods

### 2.1. Participants

A total of 149 participants accessed the online or paper-based surveys. Of the total access, 31 participants (20.8%) only partially completed the survey. The exclusion criteria for data retention were as follows: (a) participants who did not complete the survey, (b) younger than 18 and older than 100 years of age, or (c) the residents who engaged only in outdoor physical activities alone but not in groups. Thus, a total of 111 participants (68 females, 61.3% and 43 males, 38.7%), aged between 25 and 77 years (M = 53.40, SD = 14.58) completed the study. Amongst the participants, 98 (88.3%) identified themselves as being Chinese, 5 (4.5%) as Malay, 4 (3.6%) as Indian, 2 (1.8%) as Eurasian, and 2 (1.8%) as other ethnicities. The majority had a tertiary education (57.7%), and were primarily full-time employed (41.4%) or retired (24.3%). A summary of demographic details is presented in Table 1.

Participants were recruited using the snowball recruitment method from a community dwelling sample residing and engaging in gardening or outdoor activities in Singapore. The gardening group included two groups: community gardeners and individual/home gardeners. Those who engage in non-gardening outdoor activities constitute the control group for the study purposes. These three groups all engaged in activity with some form of physical activity and connection to nature elements but varied in gardening experience or social elements so that the potential contribution of these elements could be compared.

To initiate the recruitment, the study was advertised and distributed to a range of Singapore residents known to be engaged in gardening or outdoor activities. The principal investigator first approached garden leaders involved in the CIB initiative of the NParks in Singapore, and the persons-in-charge from these respective gardening groups: the Community Gardening Groups of the Community Clubs (CC), Residents’ Committees (RC), or Neighbourhood Committees (NC), the Gardening Clubs, and the volunteering gardening groups, as well as the persons-in-charge of outdoor physical activities groups of the CC, RC, or NC. Contact with respective parties was made by email, by phone, or in person. The study and the online survey link were subsequently disseminated internally to the potential participants by the persons-in-charge of the respective parties or the principal investigator. The study and the online survey link were also advertised to individual or home gardeners using posters and study advertisements published on social media platforms (e.g., Facebook) and, thereafter, through snowball recruitment of others who might be interested in the study.

Participants were asked to rate their involvement in different types of activities. Thus, the delineation of three groups was based on the self-reported involvement in different activities, as opposed to assignment of individuals to specified categories into which their activities might not have fit. Nonetheless, best efforts were made to ensure that the participants belonged to only one group, so that a cross-sectional analysis could be conducted. The residents involved in community gardening activities formed the first target group, the community gardening group. The residents who engage in gardening activities, but were individually involved in home balcony or indoor gardening instead of community gardening, formed the second target group, the individual/home gardening group. A comparison group of non-gardeners who participated in outdoor physical activities in groups formed the non-gardening (outdoor activity) control group. Before the commencement of recruitment, ethical approval was obtained from the James Cook University Human Research Ethics Committee for the proposed study (H6730).

### 2.2. Sample Size

Assuming all hypotheses were supported, an a priori power analysis for a Multivariate Analysis of Covariance (MANCOVA) with six dependent variables was conducted in G*Power to determine a sufficient sample size using an alpha of 0.05, a power of 0.95, and a small effect size (*f*_2_ = 0.20). Based on the aforementioned assumptions, an optimal sample size was indicated as 72.

### 2.3. Research Design

A cross-sectional survey research method was selected to address the research questions and was preferred over case study, naturalistic observation, or interview methods because it was more time- and cost-efficient. This cross-sectional study involved the measurement of well-being, stress and resilience levels, as well as resilience factors in community gardeners and participants of other prevalent Singapore outdoor activity pursuits to compare the potential benefits. The study variables included connection to nature, perceived stress, subjective well-being, resilience, self-esteem, optimism, and openness.

### 2.4. Measures

The survey consisted of questions relating to demographics, participants’ involvement in gardening activities and outdoor activities, participants’ self-rated connection to nature, and the original English version of psychological measures or questionnaires assessing perceived stress, subjective well-being, resilience, self-esteem, optimism, and openness. Singapore is home to a multiethnic and multilingual population with Chinese (74.3%) as the major ethnicity [147]. As English has become the home language throughout the community, the survey was first created in an English version. However, to accommodate for many others who still use Chinese as their first language, particularly those coming from Chinese backgrounds, a Chinese version of the survey was also created by using the standardized and validated Mandarin or Chinese versions of psychological measures or questionnaires. On the other hand, Malay and Tamil versions of the survey were not created due to the scarce availability of the standardized and validated Malay and Tamil versions.

#### 2.4.1. Demographics

The demographics section of the survey requested information related to participant socio-demographics (i.e., age, gender, ethnicity, education level, occupation status, duration of residency in Singapore).

#### 2.4.2. Involvement in Gardening Activities and Outdoor Physical Activities

A second section requested information regarding the participant’s involvement in gardening activities or a range of other outdoor, non-gardening physical activities, within “the past 12 months” set as the time frame for engagement. Gardening activities were listed as follows: (1) community gardening, (2) gardening at balcony, and (3) growing green plants in indoor spaces (categories 2–3 are considered individual/home gardening for the purposes of the current study). For each activity, participants used the following scale to rate the frequency of the specific gardening activity: 0 = Never, 1 = Less than once per week, 2 = Once per week or 3 = More than once per week.

The outdoor, non-gardening physical activities were listed as follows: Martial Arts (e.g., Tai Chi, Wu Shu, Malay Silat, etc.), Dancing, Brisk walking, Hiking, Jogging, Cycling, Other activities—Alone, and Other activities—In group). These outdoor, non-gardening physical activities were chosen because they are the common physical activities organized by the Community Clubs (CC), Residents’ Committees (RC), and Neighbourhood Committees (NC) that allow regular and group participation, and offer physical intensity. For the latter two items, participants were asked to specify the “other” physical activities that they had engaged in the outdoor environment, but were not listed. For all outdoor physical activity items, participants used the same frequency scale as for gardening activities. Moreover, participants used the following scale to indicate the context of engagement: 0 = Never, 1 = Alone, 2 = In group (with others), or 3 = Both (Alone and In group).

#### 2.4.3. Inclusion of Nature in Self Scale (INS; Schultz, 2001; Schultz, 2002)

The INS is a single-item visual measure assessing the construct, connection to nature. Schultz [26] advocated that the extent to which a person’s cognitive self-concept includes nature predicts the strength and closeness of the individual’s relationship with nature. To measure participants’ feelings of closeness or connection to nature, participants choose one out of seven choices, each choice with two paired circles representing self and nature, as best representing their “relationship with the natural environment.” Circle pairs range from side-by-side with no overlap (choice 1 being least inclusive) to the final pair that overlap completely and appear as one circle (choice 7 being the most inclusive), with higher scores indicating higher levels of connection to nature.

#### 2.4.4. Brief Resilience Scale (BRS)

The BRS [103,148] is a six-item measure assessing resilience as the ability to bounce back or recover from stress. Participants rate each item (e.g., “I tend to bounce back quickly after hard times”) on a five-point scale ranging from strongly disagree (1) to strongly agree (5). Three items are negatively worded, so they are reverse scored. The BRS is scored by finding the mean of the six items, with higher scores indicating higher resilience. Internal consistency has been found to be high for samples of students and patients, with Cronbach’s alphas ranging from 0.72 to 0.93 [103,149].

#### 2.4.5. Perceived Stress Scale (PSS-10)

The PSS-10 [85,150] is a 10-item measure of perceived stress. The degree to which situations in one’s life are appraised as stressful during the past month (e.g., “In the last month, how often have you felt nervous and ‘stressed’?”) is rated on a five-point scale ranging from never (0) to very often (4). Positively worded items are reverse-scored and the ratings are summed, with higher scores indicating more perceived stress. The PSS-10 has demonstrated adequate reliability and validity, with Cronbach’s alphas ranging from 0.78 to 0.83 [150,151,152] and test-retest reliability coefficients ranging from 0.55 to 0.88 [152].

#### 2.4.6. Personal Wellbeing Index—Adult (PWI-A) 

Both the original English and Chinese versions of the PWI-A as well as the user manual can be retrieved from the official website of the International Wellbeing Group [153,154]. As outlined in the manual, the PWI-A is a seven-item measure developed to measure subjective well-being. Respondents rate their satisfaction with seven life domains that are theoretically embedded: standard of living, personal health, achieving in life, personal relationships, personal safety, community-connectedness, and future security. All responses are made on an 11-point scale ranging from no satisfaction at all (0) to completely satisfied (10). The seven domain scores can be summed to yield an average score, with higher scores indicating higher subjective well-being. The PWI-A has demonstrated good internal consistency with Cronbach’s alphas ranging from 0.70 to 0.85, and good test-retest reliability across a 1–2 week interval with an intra-class correlation coefficient of 0.84 [155].

#### 2.4.7. Rosenberg Self-Esteem Scale (RSE)

The RSE [156,157] is a 10-item measure to assess self-esteem (see Appendix). Respondents rate each item (e.g., “I feel that I have a number of good qualities”) on a four-point scale ranging from strongly disagree (0) to strongly agree (3), with higher scores reflecting greater self-esteem. The RSE has demonstrated reasonable internal consistency reliability with Cronbach’s alpha at 0.77 [156] and good test-retest reliability for the two-week interval at 0.85 [158].

#### 2.4.8. Life Orientation Test-Revised (LOT-R)

The Chinese Revised Life Orientation Test was adapted by Lai et al. [159,160] from the original English version of the LOT-R. To measure optimism, the LOT-R is a 10-item measure with six scored items (three positively worded and three negatively worded) and four filler items. Respondents rate each item (e.g., “In uncertain times, I usually expect the best”) from strongly disagree (0) to strongly agree (4). Four filler items will not be scored and the negatively worded items will be reverse-scored. Consequently, only the six scored items (non-filler items) will be summed to derive an optimism score, with higher values implying higher optimism. The LOT-R exhibits reasonable internal consistency with Cronbach’s alpha at 0.78 for the entire six scored items, and test-retest reliability for an undergraduate sample over a four-month interval at 0.68 [159].

#### 2.4.9. Openness-to-Experience (English and Chinese Versions, International Personality Item Pool (IPIP))

To measure the construct of openness, the 10-item scale of the Openness-to-experience was extracted from the IPIP, which is freely accessible from the IPIP website, http://ipip.ori.org. The website is a public-domain resource that contains over 1000 personality items and over 300 scales constructed from IPIP items. Portions of the item pool have been translated to multiple languages and members of the public can contact the investigators involved with e-mail links listed at the IPIP website [161]. The Chinese version of the openness-to-experience scale was extracted from the traditional Chinese version of the 50-Item IPIP Representation of the Goldberg’s Markers for the Big-Five Factor Structure [162]. Within this 10-item measure, each + keyed item (e.g., “Believe in the importance of art”) is scored from very inaccurate (1) to very accurate (5), and each keyed item (e.g., “Avoid philosophical discussions”) is scored from very accurate (1) to very inaccurate (5). The ratings are summed, with higher scores indicating more openness to experience. According to the information at the IPIP website, this 10-item measure exhibits an acceptable level of internal consistency with Cronbach’s alpha at 0.82.

### 2.5. Procedure

The survey was constructed and hosted using Qualtrics survey software, and was also made available as a printed version for those with limited online access or not comfortable using technology. The printed version of the survey had the same content as the online version, and included both English and Chinese versions.

The study involved voluntary participation. Participants who accessed the survey online were directed to a prefacing page displaying information about the study and the voluntary nature of participation. They were also informed that choosing to proceed to the survey indicated consent and that there was an option to withdraw from the study, with directions to close the browser window. Participants who chose to complete a paper-based survey were provided with a sealable envelope in which to place their completed survey so as to retain the anonymity of their data.

### 2.6. Statistical Analyses

Data analysis was performed using IBM SPSS Statistics Version 20.0 (IBM Corp., Armonk, NY, USA) with statistical significance *p* value set to <0.05 for all analyses, without the use of Bonferroni correction as suggested by Perneger [163]. According to Perneger, Bonferroni adjustments do not assure a “prudent” interpretation of results because these adjustments are concerned with the general null hypothesis, which is irrelevant, and create more issues by increasing the likelihood of type II errors. In the present study, the data analyses were conducted in two steps: demographic analyses and main analyses. The demographic analyses compare the demographic variables across the groups to detect any potential confound. These analyses involve univariate analyses consisting of Pearson’s chi square tests and one-way Analyses of Variance (ANOVAs). The main hypotheses were tested with a one-way Multivariate Analysis of Covariance (MANCOVA) in which gardening activity served as the independent variable (Community gardening, individual/home gardening, non-gardening control), and the levels of perceived stress, subjective well-being, resilience, self-esteem, optimism, and openness served as the dependent variables. Age and connection to nature (INS score) were entered as covariates to ensure that any changes in the DVs were not due to these effects. When MANCOVA was significant, post hoc comparisons were conducted. Lastly, associations between numerical variables were analyzed using Pearson’s product-moment correlation coefficient analysis.

## 3. Results

### 3.1. Preliminary Data Screening

Prior to any analysis, preliminary data screening was conducted to check for missing or erroneous values for the fulfilment of assumptions of normality and multicollinearity. Study data were also assessed for normality distribution via visual scanning of histograms and box-plots. No correlations between dependent variables were *r* > 0.9, suggesting that multicollinearity was not of concern. The non-significant Box’s M statistics (*p* = 0.17) indicated that the assumption of homogeneity of covariance matrices was not violated.

### 3.2. Reliability of Psychological Measures

Reliability tests were run for all psychological measures used in the present study, and comparative reliabilities for published studies are presented in Table 2.

### 3.3. Socio-Demographic Characteristics

Socio-demographic characteristics of the three groups are documented in Table 1. The groups were reasonably demographically homogenous. Across all groups, the majority were Chinese and educated at the tertiary level. Amongst the community gardeners, almost half were female, and the majority were retirees or full-time employed.

Comparison analyses of participant descriptive data across the three groups were conducted. Pearson’s chi square tests showed significant differences in gender, educational level, and occupational status across the three groups, but not ethnicity. These differences were not unexpected due to the adoption of the snowballing recruitment method for participants beyond the community gardeners. An independent-samples *t*-test was conducted and it showed that there was no significant difference in PWI-A, PSS-10 and BRS scores of males and females overall, with *p* = 0.795, *p* = 0.684, and *p* = 0.302, respectively. Because of the low numbers for some of the educational levels, and occupational status categories in some of the groups, it was not possible to test for the effect of educational level, and occupational status across groups using parametric statistics.

A one-way ANOVA analysis showed that there was a significant difference across the groups on age, *F*(2, 108) = 17.57, *p* < 0.001, and INS scores, *F*(2, 108) = 11.33, *p* < 0.001. As these differences were statistically significant, these two variables were controlled for in later analyses. Post-hoc comparisons using the Bonferroni test further showed that the individual/home gardening group was significantly younger than the community gardening group (*M* = 60.20, *SD* = 13.27) and the non-gardening control group (*M* = 60.20, *SD* = 13.27), with *p* < 0.001 and *p* = 0.001, respectively. However, there was no significant difference in age between the community gardening group and the non-gardening control group, *p* < 0.397. On the other hand, post-hoc comparisons using the Bonferroni test also showed that the community gardening group reported significantly higher INS scores (*M* = 5.44, *SD* = 1.49) than the individual/home gardening group (*M* = 4.394, *SD* = 1.62), *p* = 0.009, and the non-gardening control group (*M* = 3.71, *SD* = 1.61), *p* < 0.001 respectively. However, there was no significant difference in INS scores between the individual/home gardening group and the control group, *p* = 0.25.

### 3.4. Effects of Gardening on Psychological Measures

To test Hypotheses 1 to 4 that there are between-group differences on the psychological measures, a one-way MANCOVA was performed in which gardening activity served as the independent variable (community gardening, individual gardening, non-gardening control) and the scores of PSS-10, PWI-A, BRS, RSE, LOT-R, and openness-to-experience scales served as the dependent variables, whilst controlling for age and INS scores. The results of the MANCOVA are presented in Table 3. Findings showed that there was a statistically significant main effect of gardening on the combined dependent variables, after controlling for age and INS scores, *F*(12, 202) = 2.48, *p* = 0.005; Wilk’s *Λ* = 0.760, partial *η*^2^ = 0.128.

Given the significance of the overall test, the results for the dependent variables were considered separately, using an alpha level of 0.05. As the Levene’s test of Equality of Error Variances was not significant for dependent variables except the RSE scores, which measured the level of self-esteem, the data for the RSE scores are viewed with extra caution, by using a lower alpha level *p* < 0.01 for significance. Univariate analyses showed that gardening had significant effects on the PWI-A scores, *F*(2, 108) = 5.52, *p* = 0.005, partial *η*^2^ = 0.094, and BRS scores, *F*(2, 108) = 4.18, *p* = 0.018, partial *η*^2^ = 0.073. However, gardening had no significant effect on the PSS-10 scores, *F*(2, 108) = 0.316, *p* = 0.73, partial *η*^2^ = 0.006.

With regards to the differences on the resilience factors across the three groups, univariate analyses showed that gardening had significant effects only on the LOT-R scores that measured the level of optimism, *F*(2, 108) = 4.32, *p* = 0.016, partial *η*^2^ = 0.075. Gardening had no significant difference on the RSE scores, *F*(2, 108) = 3.28, *p* = 0.041, partial *η*^2^ = 0.058, although the community gardening group reported highest RSE scores. Lastly, gardening also had no significant effect on the openness-to-experience scale scores, *F*(2, 108) = 0.715, *p* = 0.491, partial *η*^2^ = 0.013.

Post-hoc comparisons using the Bonferroni test showed that the community gardeners reported significantly higher PWI-A scores than the individual/home gardeners, *p* = 0.010 and the non-gardening control group, *p* = 0.026, respectively. However, there was no significant difference in the PWI-A scores between the individual/home gardeners and the non-gardening control group, *p* = 1.00. The community gardeners also reported significantly higher BRS scores than the control group, *p* = 0.018. While the community gardening group reported higher BRS scores than the individual/home gardening group, this difference was not significant, *p* = 1.00. Again, the BRS scores of the individual/home gardening group did not differ significantly from that of the control group, *p* = 0.118. Lastly, the community gardeners reported significantly higher LOT-R scores than the control group, *p* = 0.012. While the community gardening group reported higher LOT-R scores than the individual/home gardening group, these differences were not significant, *p* = 0.347. Again, the LOT-R scores of the individual/home gardening group did not differ significantly from the control group, *p* = 0.531.

### 3.5. Correlation Analyses

To test the remaining hypotheses, correlation analyses were run to assess whether BRS scores were significantly correlated with PWI-A and PSS-10 scores. In addition, the correlations between BRS scores and the resilience factors, the RSE, LOT-R, and openness-to-experience scale scores were also assessed. Lastly, the correlations between INS scores and BRS, RSE, LOT-R, and openness-to-experience (OTE) scale scores were also assessed. The correlation statistics are reported in Table 4.

BRS scores were found to be significantly, positively correlated with PWI-A scores, and significantly, negatively correlated with PSS-10 scores. BRS scores were also found to be significantly, positively correlated with RSE scores, LOT-R scores, and openness-to-experience scale scores. These correlations between resilience and the respective resilience factors suggest that the factors are associated constructs but do not measure the same construct, which is consistent with the literature review.

In summary, there are significant differences in age and INS scores (i.e., the measure of connection to nature) across the three groups. Taking these inherent between-group differences into account, gardening was found to have a significant main effect on some of the mental health benefits such as subjective well-being. Specifically, the community gardening group reported highest scores of PWI-A (i.e., the measure of subjective well-being) across the three groups. The community gardening group also reported higher BRS scores (i.e., the measure of resilience) and higher LOT-R scores (i.e., the measure of optimism) than the non-gardening control group. Besides that, the engagement in individual/home gardening activities and in the non-gardening outdoor activities did not appear to have a significant impact on the scores of the psychological measures of subjective well-being, perceived stress, resilience, and resilience factors (i.e., self-esteem, optimism, and openness). Lastly, correlation analyses showed that BRS scores were positively correlated with PWI-A scores and negatively correlated with PSS-10 scores. Moreover, BRS scores were also positively correlated with the resilience factors, the RSE, LOT-R, and openness-to-experience scale scores. INS scores were also positively correlated with BRS, RSE, LOT-R, and openness-to-experience scale scores.

In light of the data of the present study, the following hypotheses were supported:

**Hypothesis 1** **(H1).** *Community gardeners reported significantly higher levels of subjective well-being than the individual/home gardeners and the non-gardening control group.*

**Hypothesis 3** **(H3).** *Resilience levels were significantly different across the community gardeners, the individual/home gardeners, and the non-gardening control group.*

**Hypothesis 4b** **(H4b).** *The levels of optimism were significantly different across the community gardeners, the individual/home gardeners, and the non-gardening control group.*

**Hypothesis 5** **(H5).** *Resilience was positively correlated with subjective well-being.*

**Hypothesis 6** **(H6).** *Resilience was negatively correlated with perceived stress.*

**Hypothesis 7** **(H7).** *Resilience was positively correlated with each of the individual resilience factors (i.e., self-esteem, optimism, and openness).*

**Hypothesis 8** **(H8).** *Community gardeners reported significantly higher levels of connection to nature (i.e., the INS score) than the individual/home gardeners and the non-gardening control group.*

**Hypothesis 9** **(H9).** *Connection to nature (i.e., the INS score) was positively correlated with resilience.*

**Hypothesis 10** **(H10).** *Connection to nature (i.e., the INS score) was positively correlated with each of the individual resilience factors (i.e., self-esteem, optimism, and openness).*

## 4. Discussion

We first examine and discuss the key findings reported here in the context of the hypotheses and past research findings. Subsequently, the clinical implications and the limitations of the study, as well as future recommendations are presented.

Through the data gathered from the survey, the present study found evidence in terms of quantitative data for a number of valuable findings. Firstly, consistent with hypothesis 1 (Hypothesis 1), after controlling for the effects of age and connection to nature, the community gardening group reported significantly higher levels of subjective well-being than the individual/home gardening and the non-gardening control groups, with an indication that community gardening activities are superior to individual/home gardening or non-gardening outdoor activities. This superiority is a unique result compared to the past research findings merely documenting the relationship between community gardening and well-being [17,81]. The present study is also different from most of the previous studies in Western countries because it also considered the potential contribution of the individual elements of community gardening on the levels of subjective well-being by comparing to individual/home gardening or non-gardening outdoor activities. It should be noted that the three groups investigated in the present study all engaged in physical activities involving some exposure to nature but varied in gardening experience or social elements. The community gardening group engaged in activities covering physical activity, connection to nature, gardening experience, and social elements compared with the individual/home gardening group in which the social element of gardening was absent, and the non-gardening control group in which the gardening element was absent. As there is no statistically significant difference between the individual/home gardening group and the non-gardening control group, higher levels of subjective well-being reported by the community gardeners likely stemmed from the combined effects of the physical activity, contact or connection to nature, gardening experiences and social elements.

Contrary to Hypothesis 2, there was no significant difference in the levels of perceived stress across the three groups, after controlling for age and the levels of connection to nature. Although the levels of perceived stress tended to be lower for the community gardening group as compared to the other two groups, this difference might only be considered at the level of practical significance if supported in future research of a qualitative nature. These current findings varied from the past studies with qualitative data [57,81], which advocated that taking part in community gardening helped relieve stress. However, the current findings are consistent with a past study with quantitative data [91], which also found no significant difference in the perceived stress levels between allotment gardeners, home gardeners and those who performed outdoor physical activity. The current findings might be explained by the restorative effects of the contact or connection to nature element, which was also a common element across the groups. Participants across these three groups at least had certain levels of connection to the nature, which would be helpful for the attenuation of deliberate attention through restoration processes that are affected by stress. Further, their activities in nature could also help disrupt the negative stress process [13,14,22], resulting in no significant differences in levels of perceived stress across the groups. It could also be argued that the stress reduction effect of community gardening in the present study was non-significant because of the varying baselines of the perceived stress levels of each participant. Thus, the pre- and post-testing design might be considered to more effectively capture the change of stress reduction of engagement in community gardening for each participant. Lastly, despite no significant difference in perceived stress levels across the three groups, the community gardening group still reported the highest levels of subjective well-being, suggesting evidence for the mental health benefits of the community gardening in the Singapore context.

Aligned with Hypothesis 3, resilience levels were significantly different across the groups, after controlling for age and levels of connection to nature. In particular, the community gardening group reported significantly higher resilience levels than the non-gardening control group, but not the individual/home gardening group. Given that the community gardening group engaged in activities covering physical activity, connection to nature, gardening experience and social elements but the individual/home gardening group in which the social element of gardening was absent, and the non-gardening control group in which the gardening element was absent, these findings were expected. It could be argued that the combined effects of the gardening experiences and social elements of the community gardening group could have multiplied and overshadowed the individual effect of the social element of the non-gardening control group. However, the combined effects did not overshadow the individual effect of the gardening experiences element of the individual/home gardening group, suggesting gardening experience played a bigger role than the social element in the proportions of contribution on resilience levels.

Consistent with Hypothesis 5 and Hypothesis 6, correlation analyses show that resilience levels were positively correlated with levels of subjective well-being and negatively correlated with levels of perceived stress, aligning with past research findings [108]. The overall results shed light onto the positive influence of community gardening on resilience levels, and suggest that there is some association between resilience levels and separate elements like gardening experience or social elements.

Concerning the resilience factors, and consistent with Hypothesis 7, correlation analyses also showed that resilience levels were positively correlated with each of the resilience factors, and levels of self-esteem, optimism and openness. However, contrary to Hypothesis 4.a., no significant difference in levels of self-esteem across the three groups was observed in the study. The current findings thereby differ from those reported by [82] in which the non-gardeners were recruited from local supermarkets. Wood et al. did not specify whether the non-gardeners in their study engaged in regular physical outdoor activities, so they are potentially different from the non-gardening group of the present study in which the control group engaged in regular, non-gardening, physical outdoor activities. Aligned with Hypothesis 4.b., the levels of optimism were significantly different across the three groups, with significantly higher levels of optimism were observed in the community gardening group than the non-gardening control group. This finding provides a valuable expansion to the existing literature pertaining to optimism [133] by indicating that community gardening is linked with higher levels of optimism, which in turn potentially enhances the community gardeners’ resilience potentials. Lastly, contrary to Hypothesis 4.c., no significant difference on the levels of openness was observed across three groups in the present study although openness was found to be positively correlated with resilience levels. This finding is unique, in that prior studies have not included openness to experience as a factor of interest, and it therefore helps to expand the literature in terms of resilience and openness, which is one of the resilience factors.

Concerning contact and connection to nature, the present study differs from those in the past (e.g., [91]) wherein it was assumed that community gardeners had the most engagement with nature during their gardening activity. Instead, the present study measured connection to nature by using the INS scores. Consistent with Hypothesis 8, the community gardening group reported significantly higher levels of connection to nature (i.e., the highest INS score) than the individual/home gardening and the non-gardening control groups. It could be argued that the community gardeners rated themselves as having more connection to nature because they not only have direct contact with the plants they cultivated, but the community garden also provides expanded space and greenery features. This is in comparison to the home gardeners who only have direct contact with the plants they cultivated, and those non-gardeners who engaged in physical activities in outdoor environments who only have contact with green landscapes.

Consistent with Hypothesis 9 and Hypothesis 10, correlation analyses showed that connection to nature was also positively correlated with resilience, whereby similar patterns were observed between levels of connection to nature and each of the resilience factors. This is especially salient for the possible explanation for the benefits of community gardening on resilience levels. In view of the findings, community gardening, particularly in a city-state with spatial constraints such as Singapore, has the potential to help residents to enhance their mental health, through connecting to nature and offsetting the ongoing loss of human-nature interactions [164].

### 4.1. Clinical Implications of the Present Study

Based on the key findings of this study, there are several valuable clinical implications. Firstly, the present study validated the contribution of community gardening to subjective well-being, which is a vital aspect of mental health. The findings of the present study, by uniquely comparing across two gardening groups together with a non-gardening control group, further imply that the effect of community gardening is significantly superior to individual/home gardening and non-gardening. This is possibly due to the more powerful, combined effects of the physical activity, connection to nature, gardening experiences, and social elements integrated into community gardening activities.

Secondly, the present study findings presented here fill in the existing literature gap by highlighting the unique implications of community gardening on the participants’ resilience potentials. The present study was the first to offer quantitative data to show that community gardening is linked with higher levels of resilience than with other non-gardening, outdoor social activities. Given the resilience-stress relationship, whereby resilience is frequently discussed as an aspect of adaptive coping and mental health [108] and is also documented to be trainable [165], the study’s findings could help to enhance resilience levels of the residents who are at risk for high stress. Specifically, the findings could be used to guide healthcare policies to implement programs designed to enhance resilience and to ameliorate the risk factors to mental health. As stress is common in the current, fast-paced metropolitan society and its negative effects on mental health are indubitable, health care practitioners and community service providers could draw on the growing evidence to introduce community gardening as a useful mental health promotion strategy and encourage people to participate in community gardening activities.

Thirdly, the present study also expands the existing literature by investigating how community gardening is individually associated with resilience factors, self-esteem, optimism, and openness. To further supplement the findings by [123], the present study distinguished between individual and community gardening as separate aspects. Although no significant difference in self-esteem was found across the groups, the community gardening group was observed to have the highest level of self-esteem. Being the first study that contributed quantitative data assessing optimism levels and resilience amongst community gardeners, the current study findings support the associations between optimism and resilience [117]. Moreover, the community gardeners recorded higher levels of optimism than the non-gardeners, which further expands the existing literature, extending the mental health benefits of community gardening to optimism and resilience potentials. In addition, being the first study that examined the associations among openness, resilience, and community gardening, these findings make a valuable contribution to the existing literature, albeit if only to confirm that there was no significant differences in openness across the groups. In view of the relationships between community gardening and three resilience factors respectively, the study findings can help guide healthcare professionals to incorporate community gardening into mental health programs or psychological interventions.

Lastly, the present study is unique to the extent that it is the first study to investigate the mental health benefits of community gardening in the Singapore context, which is Eastern-oriented as opposed to the predominance of findings from Western samples. With an ever-increasing spatial constraint, promoting public mental health in cities is a focal challenge. The study findings confirm that the current Singapore community garden movements indeed have potential for sustainable benefits to the mental health of residents in Singapore in terms of subjective well-being and resilience. The improved knowledge on subjective well-being, perceived stress and resilience, as well as the resilience factors, can better guide researchers and clinicians in developing plausible, sustainable strategies to enhance mental health benefits. Besides that, community gardening, which integrates empowerment and social elements, could also be included as part of the therapy in primary care settings or for those with mild emotional distress in Singapore.

### 4.2. Limitations of the Present Study

Despite the best efforts when designing the present study, there are several limitations that should be highlighted. Firstly, since the present study employed a cross-sectional design, causal inferences between community gardening and the levels of subjective well-being, perceived stress, and resilience should not be implied [166]. It is possible that individuals who engage in community gardening have more time to commit to this activity (most of the current sample of community gardeners was retirees), which could result in more powerful combined effects on the mental health benefits. Besides that, while participants in all three groups engaged in activities covering physical activity elements, a measure of the duration spent on each activity may be helpful in examining another moderator of this relationship.

Secondly, the sample profile was in a way restricted by the snowballing recruitment method and considered as a representative only of the particular subgroup of people sampled because the sampling coverage is restricted to the contact circles of a particular group of people. This method could have inhibited participation by those individuals who have no access to the internet or technology. Consequently, the sampled population may have a tendency to under-represent those with limited online access or who are not comfortable using technology. To reduce this potential, a printed version was made available, but it was accessible only to those with whom the researcher was able to reach through recruitment advertisements. As there is no single general website for all of the community gardening groups under the CIB initiatives, the principal investigator needed to approach the garden leaders of selected gardening groups, and thereafter snowballing to others who might be interested in the study. Consequently, uneven group sizes might have limited the power of the current study. Besides that, the individual/home gardeners were significantly younger than the other two groups. However, the present study had controlled for age in the analyses. Future studies may consider examining the resilience potentials in closer detail across age ranges.

Lastly, another concern is the extent to the truthfulness of the responses. Survey questionnaires are generally prone to social desirability bias [166], but the survey method is still relevant and applicable because the present study targeted to assess respondents’ perceptions and judgements (e.g., perceived stress levels), instead of the quantifiable behaviors. To further address this limitation, future studies might consider to gather additional corroborative information by adding clinical interviews. Paulhus [167] reported that respondents tended to report more desirable responses when they were requested to put identifying information (e.g., name) on the questionnaire than when told not to. Consequently, in order to reduce social desirability bias in the present study, participants were clearly advised that all responses would remain anonymous. Moreover, to reduce response bias, psychological measures with negatively worded items were selected, and their good reliabilities in the present study exclude this as a critical limitation.

Notwithstanding the above limitations, the present study widens the scope of mental health benefits of community gardening by examining resilience potentials. Studying resilience factors across the three groups provides an exclusive contribution to the mental health benefits of community gardening research within an Eastern-oriented context.

### 4.3. Recommendations for Future Research

It could be prudent to follow up on the current findings with a longitudinal design to track and compare the pre- and post-levels of subjective well-being, perceived stress, and resilience across groups. Moreover, future studies could employ different analyses to examine the mediating or moderating role of the resilience factors on the relationships between community gardening and mental health benefits. Future studies could also look at differentiating adolescent, adult, and elderly individuals who engage in community gardening for direct comparison purposes. Improved knowledge on the benefits of community gardening across these age groups may help policy makers and horticultural therapists decide the direction for the promotion of gardening activity.

## 5. Conclusions

The present study provides a novel understanding of the mental health benefits of community gardening within the context of Singapore, a high-density urbanized Southeast Asian city-state. The findings suggest that, despite similar levels of perceived stress, the community gardeners demonstrated higher levels of subjective well-being than the individual/home gardening group and the non-gardening control group, indicating the superior effects of community gardening on subjective well-being, a vital aspect of mental health benefits. These novel findings pertaining to community gardening as well as its relationship with resilience levels fill in some of the gaps in the relevant research literature. Moreover, the association between community gardening and optimism levels also provides evidence for the ability of community gardening to enhance resilience potentials, which can help individuals to adapt well to stressful situations. The present study also provides evidence for the notion that community gardening, along with other types of green landscape exposures, can enable residents to connect with nature. This in turn appears to enhance the mental health of community gardeners. Since community gardening is linked with evident mental health benefits, despite the limitations of this study, the additional values of community gardening and its combined effects of physical activity, nature exposure, gardening experiences, and social elements is worth noting. More confirmatory studies or longitudinal studies are needed to examine the feasibility of developing psychological interventions tapping on community gardening.

## Figures and Tables

**Table 1:** Demographic Characteristics (Means (SD), No. (%)) Across Three Groups.

Variable	Participant Group		
	Non-Gardening Control (*n* = 28)	Individual/Home Gardening (*n* = 38)	Community Gardening (*n* = 45)
*M* Age in years	55.54 (11.62)	43.76 (12.99)	60.20 (13.27)
Age range	36–75	25–73	25–77
Gender: Female	16 (57.1%)	32 (84.2%)	20 (44.4%)
Ethnicity: Chinese	23 (82.1%)	35 (92.1%)	40 (88.9%)
Education Level: Tertiary	13 (46.4%)	33 (86.8%)	18 (40.0%)
Occupation Status: Full-time employed	14 (50.0%)	22 (57.9%)	10 (22.2%)
Duration of Residency in Singapore (≥2 years)	28 (100.0%)	36 (94.7%)	44 (97.8%)

**Table 2:** Cronbach’s Alphas for the Psychological Measures.

Measures	Published Cronbach’s Alphas	Cronbach’s Alphas for the Present Study	Qualitative Reliability
BRS	0.72–0.93	0.85	Good
PSS-10	0.78–0.83	0.86	Good
PWI-A	0.70–0.85	0.88	Good
RSE	0.77	0.85	Good
LOT-R	0.78	0.71	Acceptable
Openness to Experience	0.82	0.80	Good

Note: BRS = Brief Resilience Scale, PSS-10 = Perceived Stress Scale, PWI-A = Personal Wellbeing Index, RSE = Rosenberg Self-Esteem scale, LOT-R = Life Orientation Test-Revised.

**Table 3:** Means (SD) of Study Variables and MANCOVA Results of Differences Across Three Groups While Controlling for Age and Inclusion of Nature in Self (INS) score.

Variable	Participant Group			*F* (2, 108)
	Non-Gardening Control (*n* = 28)	Individual/Home Gardening (*n* = 38)	Community Gardening (*n* = 45)	
PWI-A	7.03 (0.78) ^b^	6.98 (1.21) ^b^	8.17 (1.06) ^a^	5.52 **
PSS-10	14.21 (5.16)	15.47 (6.07)	11.42 (6.40)	0.32
BRS	3.01 (0.78) ^b^	3.47 (0.62)	3.74 (.68) ^a^	4.18 *
RSE	18.50 (2.80)	19.82 (4.38)	22.31 (4.12)	3.28
LOT-R	13.75 (2.44) ^b^	14.68 (3.08)	16.84 (3.53) ^a^	4.32 *
Openness-to-experience	32.11 (6.68)	35.18 (4.83)	34.69 (6.36)	0.72

Note. * *p* < 0.05; ** *p* < 0.01. Post-hoc analyses indicate that ^a^ group differs statistically significantly from ^b^ group/s. PWI-A = Personal Wellbeing Index, PSS-10 = Perceived Stress Scale, BRS = Brief Resilience Scale, RSE = Rosenberg Self-Esteem scale, LOT-R = Life Orientation Test-Revised.

**Table 4:** Pearson’s Correlation Statistics for Study Variables.

Variable	PWI-A	PSS-10	BRS	RSE	LOT-R	OTE	INS
PWI-A		−0.571 ***	0.467 ***	0.548 ***	0.340 ***	0.167	0.365 ***
PSS-10			−0.519 ***	−0.608 ***	−0.475 ***	−0.123	−0.381 ***
BRS				0.595 ***	0.393 ***	0.309 ***	0.424 ***
RSE					0.448 ***	0.312 ***	0.398 ***
LOT-R						0.276 **	0.300 ***
OTE							0.242 **
INS							

Notes. *n* = 111; ** *p* < 0.01, *** *p* < 0.001. PWI-A = Personal Wellbeing Index, PSS-10 = Perceived Stress Scale, BRS = Brief Resilience Scale, RSE = Rosenberg Self-Esteem scale, LOT-R = Life Orientation Test-Revised, OTE = Openness to experience, INS = Inclusion of nature in self.
